# Views on Workplace Policies and its Impact on Health-Related Quality of Life During Coronavirus Disease (COVID-19) Pandemic: Cross-Sectional Survey of Employees

**DOI:** 10.34172/ijhpm.2020.127

**Published:** 2020-08-03

**Authors:** Eliza Lai-Yi Wong, Kai-Fai Ho, Samuel Yeung-Shan Wong, Annie Wai-Ling Cheung, Peter Sen-Yung Yau, Dong Dong, Eng-Kiong Yeoh

**Affiliations:** JC School of Public Health and Primary Care, Faculty of Medicine, The Chinese University of Hong Kong, Hong Kong SAR, China.

**Keywords:** Workplace Policies, Health-Related Quality of Health, Occupational Health, Employees, COVID-19, Hong Kong

## Abstract

**Background:** This study explored the degree of views towards supportive workplace policies among employees during coronavirus disease 2019 (COVID-19) pandemic and its association with health-related quality of life (HRQoL) in Hong Kong.

**Methods:** A cross-sectional study was conducted in 1049 employees using online self-administered questionnaire. Views on workplace policies were measured in term of agreement on its comprehensiveness, timeliness and transparency whereas HRQoL was measured using EQ-5D-5L Hong Kong version. Univariate estimates on the impact of HRQoL from views of measures in workplace was done. Qualitative comments on the suggestions to strengthen workplace measures were collected and presented descriptively.

**Results:** Of 1048 respondents, 16% reported that no workplace measures nor guidelines were existed in their company related to the COVID-19 pandemics. Those who reported having workplace policy were not satisfied with the arrangement in term of comprehensiveness (36%), timeliness (38%), and transparency (63%). Regarding to the policy measure, only 68% respondents reported that their workplace supplied face masks to them. The health index was 0897, which was lower than the norm of 0.924. 64% of respondents reported having a health problem in at least 1 of 5 dimension of EQ-5D-5L with the highest proportion of having problem in anxiety/depression (55%). In addition, the workplace policy and measure had a direct effect of 0.131 on health outcome. Perception of infection risk had a direct effect of 0.218 on health outcome and partly mediated the relationship between workplace policy and measure and health outcome (0.066).

**Conclusion:** The study highlighted the workplace policy and measure is an important mean to minimize infection risk at workplace so as to reduce tremendous stress and health outcome caused by a COVID-19 pandemic. Workplace measures related to COVID-19 pandemic should be further strengthen to mitigate the risk of infection and protect employee’s health.

## Background

**Key Messages**

** Implications for policy makers**

- Our study highlighted the discrepancy of workplace policy to protect their health in term of comprehensiveness, timeliness and transparency. The proportion of dissatisfaction with workplace policy was significantly higher in blue-collar group and associated professional group.
- Evident discrepancy views in workplace measures have contributed to a significant amount on employee’s health during coronavirus disease 2019 (COVID-19). The present study highlighted the negative impact on the health-related quality of life associated with the lack of workplace policies, lack of protective equipment supply and dissatisfied with workplace policies. The dimension of anxiety and depression was identified as significantly worse than other dimensions of quality of life.
- Based on the evidence, government and organization should set up specific strategies to ensure adequate supply of protective equipment (eg, facemask) and to enforce sufficient measures in the workplace (social/physical distancing, home office with technology support) as well as.

** Implications for the public**

Effective public health strategies to mitigate the risk of infection of coronavirus disease 2019 (COVID-19), policy at the individual level and organization level are equally important. This study may assist the organization to engage employee in reviewing and strengthening the workplace measures in order to enhance occupational safety and protect health in working population. The organization will also gain a better level of understanding on the need of employee in order to relieve their worry about being infected with COVID-19 in their workplace. The results highlighted the importance role of government for strengthening workplace policy at organization level.

As of March 9, 2020, coronavirus disease 2019 (COVID-19) has spread across more than 110 countries after the initial case identified at the end of December 2019 in Wuhan, China with 113 702 confirmed cases and 4012 deaths.^1^ On March 11, 2020, the World Health Organization (WHO) declared the COVID-19 outbreak as pandemic^2^ and it is a serious concern for public health. Evidence highlighted social mobilization plays a significant in the infectious disease spread.^3^ In order to mitigate the rapid spread of COVID-19 through international contact and outbreak at local community, many jurisdictions have implemented policy interventions and public health measures to minimize the spread of COVID-19. The Government of the Hong Kong Special Administrative Region (HK) announced the first confirmed case on January 23, 2020 and the number increased to 129 cases by March 10, 2020. The response level under the ‘Preparedness and Response Plan for Novel Infectious Disease of Public Health Significance’ (the Preparedness and Response Plan) was raised to emergency response level since January 26, 2020. In addition, a series of policies and guidance were established such as on border control, facility closure and quarantine guidelines. Subsequently, the government asked all its employees (except those providing essential/emergency services) to ‘work from home’ from January 28, 2020. The announcement came in the wake of similar measures in other majority industries such as education, social service units including non-governmental organizations, and commercial offices in private sectors to adopt home offices or flexible work arrangements.

A survey reported that more than 80% HK companies had implemented work from home arrangements,^4^ but the extents were not company-wide and varied in difference industries. A proportion of companies also did not follow these at all. HK changes from vivid and energetic metropolis to static city with limited retail and catering service overnight. With increasing opinions related to economic stress in favour of service resumption, the government announced resumption of office duty on February 27, 2020, effective by roster system on March 2, 2020 after 33 days of special work arrangement at home.^5,6^ Meanwhile, the psychological stress may increase due to the fear of infection after resuming work. The WHO has provided a series of guidelines for protection for both, health and non-health workers.^7^ This workplace guidance also includes the information on coronavirus, cleaning the infrastructure, face masks, management of confirmed COVID-19 cases, travelling and meetings, and certifying absence.^8^ Thus, implementing appropriate workplace guideline and assessment the efficacy of those guideline in working population as part of epidemic preparedness are important in regions with elevated outbreak risk in order to identify existing gaps and improve occupational safety and viral surveillance.

In the experience of severe acute respiratory syndrome (SARS), actual physical health danger and new psychological problem related to self-perceived likelihood of infection may appear as 2 diseases looming during infectious outbreak.^9^ According to the availability heuristics, personal judgement of likelihood of risk is biased by the ease of recall from memory of experience,^10^ heritage values in the cultural context,^9^ and dissemination of information in any means and sources.^9^ Perception of risk consequently brings out different levels of psychological stress which may hinder well-being and lead to different choices of coping action to the infectious disease outbreak.^11^ Besides stress, limited study has explored the impact of perceived risk on well-being; only one study has found that life stress inversely associated to physical and mental health.^12^ The workplace policy is an important tool and resource to protect health and support healthy behaviours in workplace which may ease one’s adaption to perceived risk of infection in the workplace. However, workplace policy was frequently modified because of the evolving understanding of COVID-19. In this context, it is therefore essential to understand the role of workplace policy and well-being of employees after work resumption, but no study to date has examined the interplay role between workplace policy and perceived risk on health outcome in employees.

###  Study Aim and Hypotheses

**Figure 1:**
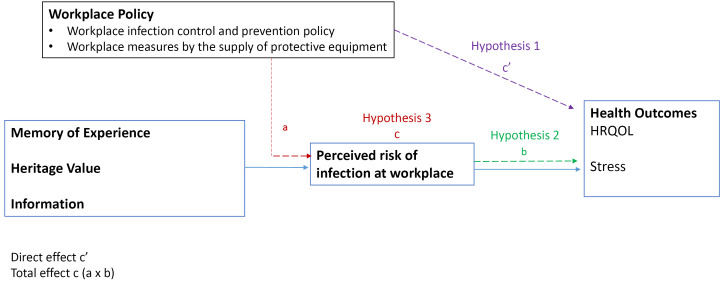
Research Model With 3 Hypothesis. Abbreviation: HRQoL, health-related quality of life.

This study explores the relationship between employee’s view on workplace policy, perceived likelihood of risk and health outcome in working population during COVID-19 pandemic. First, we hypothesized that those employees who are more satisfied with workplace policy and measure would have better health outcome (hypothesis 1). Second, we hypothesized lower perception of infection risk at the workplace associated with better health outcome (hypothesis 2). Third, we further hypothesized that higher satisfaction on workplace policy will alleviate the negative path between perceived risk and health outcome (hypothesis 3). The research model is shown in Figure 1.

## Methods

###  Study Design

 An anonymous cross-sectional survey was conducted on an online platform (Google Form) from 17 to February 27, 2020 in HK. Those who were HK residents, aged 18 years or above, working either on a full- or part-time basis, employed or self-employed, and able to understand Chinese were eligible for the survey, whereas those who were retired, housewives, and students were excluded.

###  Data Collection

 Respondents were recruited using convenience sampling methods and the participation was voluntary and anonymous. Upon the survey, an electronic informed consent was obtained. The collected data were stored in Google Drive and password-protected to ensure data security.

###  Instruments

The questionnaire consisted of 6 sections: (1) views on workplace infection control and prevention policy and guideline in terms of comprehensive, timely, and transparency using 3-point scale (not at all, some extent, good enough); (2) availability of workplace measure by the supply of protective equipment in their companies using binary response (yes, no); (3) perceived risk of infection of COVID-19 due to the work using 5-point scale (very worried to not at all); (4) health-related quality of life (HRQoL) using EQ-5D-5L HK version; (5) demographics; and (6) open text for suggestion to enhance workplace protection. The culturally validated HK Chinese version of the EQ-5D-5L instrument (EQ-5D-5L HK) was adopted to assess the health outcome.^13-15^ The instrument consists of a 5-dimension descriptive system (mobility, self-care, usual activities, pain/discomfort, and anxiety/depression). Each dimension has 5 response levels describing the health status with a level of severity (no problem, slight, moderate, severe, and unable/extreme problems). Once the health status was assessed from the description part, the 5-digit number can be converted into a single preference weight health index score ranging from 0 (dead) - 1 (full health) using the valuation of HK population tariff to reflect the HRQoL.^15^

###  Statistical Analysis

Data management and analysis were conducted using R version 4.0.0. Descriptive information including the demographics of respondents, the views on the workplace policy (grouped as binary variable), workplace measure by supply of protective equipment and perceived risk of infection. Assuming different responses would be found among the employee working in different occupational groups, Chi-square/Fisher exact test and Kruskal-Wallis test were used to show the differences. For the self-reported health outcome, 5-dimension scores were translated to a single health index score to reflect the HRQoL. The health status in each of 5 dimensions was presented in a binary way (with problem and without problem). A series of concurrent regression analysis was conducted separately for the health outcomes including HRQoL and health status in 5 dimensions. The purpose was to examine if individuals who were less satisfied with workplace policy (X), would be more likely to perceive higher infection risk at workplace (M), and if this would in turn lead to lower health index score (Y), which may result in worsening health outcomes and HRQoL. The first cluster of correlates included workplace policy and health outcomes (regression 1). The second cluster of correlates was perceived infection risk at workplace and health outcomes (regression 2). The third cluster of correlates was workplace policy and health outcome x perceived risk (regression 3). The mediation analysis using structural equation modelling was comprised of above 3 regressions to determine the interplay of the perceived risk of infection at workplace between workplace policy and measure and the health outcome. If there was no effect of workplace policy and measure on health outcome, perceived risk of infection at workplace fully mediated in the pathway (full mediation model). If there was an effect of workplace policy and measure on health outcome, perceived risk of infection at workplace partially mediated in the pathway (partial mediation model). Those demographics correlated with health outcomes (HRQoL) were included for adjustment in the model. Goodness of fit of the model was evaluated based on the goodness of fit index (GFI), adjusted GFI (AGFI), normed-fit index (NFI), comparative fit index (CFI), Tucker-Lewis Index (TLI) and root mean square error of approximation (RMSEA). The GFI, AGFI, NFI, CFI and TLI should achieve values of ≥0.90 while RMSEA should less than 0.08 respectively if the model fits the data well.^16,17^ Alpha levels of *P* < .050 were specified as the threshold to indicate statistical significance.

## Results

###  Demographics

In total, 1196 respondents responded to the survey. Of them, 148 respondents reported that they were retired or unemployed, or did not provided their work titles; therefore, only 1048 (88%) valid responses retained in the data analysis. Of the 1048 respondents, most of them were aged 40-49 years (33%), female (68%), with highest level of education attained were university graduate or post-graduate (89%), married (53%) and living with family (93%). For the employment status, a majority worked on a full-time basis (91%) and in the field of ‘Professionals’ (43%) (Table 1). According to the HK working population statistics,^18^ the recruited sample had more women, elder and fewer respondents from the occupational groups of blue-collar workers (service workers/sales/craft workers, plat/machine operators and assemblers, elementary workers, and others). Hence, the last 4 occupation groups were grouped as blue-collar workers and a weighed adjustment was applied to the occupational groups of study population to more accurately reflect the working population in HK.

**Table 1:** Characteristics of the Participants

	**Study Sample (n = 1048), No. (%)**	**HK Population** ^a^ ** (%)**
Age group^b^		
18-29	217 (20.7)	(18.6)
30-39	294 (28.1)	(25.0)
40-49	345 (32.9)	(23.6)
50-59	163 (15.6)	(22.7)
≥60	28 (2.8)	(10.1)
Gender		
Female	712 (67.9)	(50.5)
Male	336 (32.1)	(49.5)
Education level		
Lower secondary or below	10 (1.0)	
Upper secondary	103 (9.8)	
Form 6 to form 7	148 (14.1)	
University or above	787 (75.1)	
Marital status		
Single	444 (42.4)	
Married/cohabited	557 (53.1)	
Divorced/widowed	47 (4.5)	
Living status		
Alone	68 (6.5)	
Lived with family/others	980 (93.5)	
Employment status		
Full-time	950 (90.7)	
Part-time	98 (9.4)	
Occupation group		
Managers and administrators	183 (17.5)	11.6
Professionals	440 (43.0)	7.9
Associate professionals	256 (24.4)	20.6
Clerical support workers	104 (10.9)	12.8
Service and sales workers	53 (5.1)	16.0
Craft and related workers	2 (0.2)	6.3
Plant and machine operators and assemblers	0 (0.0)	4.4
Elementary occupations	3 (0.3)	20.2
Others^c^	7 (0.7)	0.1
Industrial field		
Manufacturing	35 (3.3)	2.7
Construction	105 (10.0)	9.1
Import/export and wholesale	100 (9.5)	11.5
Retail, accommodation and food services	28 (2.7)	16.3
Transportation, storage, postal and courier services, Information and communications	100 (9.5)	11.7
Financing, insurance, real estate, professional and business services	116 (11.1)	20.5
Public administration, social work activities and personal services	526 (53.6)	27.7
Others^d^	2 (0.2)	0.6
Self-report having chronic illness		
Yes	151 (14.4)	
No	897 (85.6)	

^a^Hong Kong Annual Digest of Statistics 2019 – Overall labor force statistics. Census and Statistics Department, HKSAR, 2019. ^b^Data for HK Census and Statistics Department refers to population ≥15 years and for study sample ≥18 years. ^c^Others: Farm workers, Animal husbandry workers and Fishermen, Occupations unidentifiable or inadequately described. ^d^Others: Agriculture; forestry and fishing; Mining and quarrying; Electricity and gas supply; Water supply; sewerage, waste management and remediation activities and industrial activities unidentifiable or inadequately described.

###  Views on Supportive Infection Control and Prevention Policy and Measure in the Workplace

Regarding the supportive policy and measures regarding COVID-19 in the workplace, 84% respondents reported different extents of workplace policies in place. Those who reported that their company lacked any policy were mainly in the blue-collar group (23%, *P* < .001) and 4 industrial sectors: import/export, wholesale, and retail trades (18%); manufacturing (17%); miscellaneous social and personal services (16%); and accommodation and food services (11%) (*P* < .001). Those who reported having workplace policy in their company were not satisfied with the arrangement and provided negative comments on its comprehensiveness (36%), timeliness (38%), and transparency (63%). The comprehensiveness of workplace policy was perceived as being inadequate significantly more by the associated professional group (41%) and the blue-collar groups (40%) than the other 3 occupational groups (*P* = .004). Again, the associated professional group (44%) and the blue-collar groups (42%) also had significantly worse experience than the other 3 occupational groups about the timely updates on workplace policy (*P* = .002). For the transparency, there was no significant (*P* = .461) among the occupational groups (Table 2).

**Table 2:** Views Towards Workplace Policy by Occupation Groups

	**Managers and Administrator** **No. (%)**	**Professionals** **No. (%)**	**Associate Professionals** **No. (%)**	**Service/Shop Sales Workers** **No. (%)**	**Blue-Collar Workers** ^a^ **No. (%)**	**Total** **No. (%)**	***P*** **Value** ^b^
	**[n = 121]**	**[n = 82]**	**[n = 216]**	**[n = 134]**	**[n = 493]**	**[n = 1048]**	
Workplace policy in place							<.001
Yes	107 (88.0)	75 (91.4)	200 (92.6)	119 (88.5)	379 (76.9)	881 (84.1)	
No	15 (12.0)	7 (8.6)	16 (7.4)	16 (11.5)	114 (23.1)	167 (15.9)	
	**[n = 107]**	**[n = 75]**	**[n = 200]**	**[n = 119]**	**[n = 379]**	**[n = 880])**	
Comprehensiveness							.004
Negative	25 (23.6)	23 (30.6)	83 (41.4)	36 (30.4)	152 (40.0)	319 (36.2)	
Positive	82 (76.4)	52 (69.4)	117 (58.7)	83 (69.6)	228 (60.0)	562 (63.8)	
Timeliness							.002
Negative	27 (25.5)	23 (31.1)	89 (44.3)	39 (32.6)	159 (42.0)	337 (38.3)	
Positive	80 (74.4)	52 (68.9)	111 (55.7)	80 (67.4)	220 (58.0)	543 (61.7)	
Transparency							.461
Negative	62 (57.8)	47 (62.7)	135 (67.5)	79 (66.3)	235 (62.0)	558 (63.4)	
Positive	45 (42.2)	28 (37.3)	65 (32.5)	40 (33.7)	144 (38.0)	323 (36.6)	
	**[n = 121]**	**[n = 82]**	**[n = 216]**	**[n = 134]**	**[n = 493]**	**[n = 1048]**	
Supply of protective equipment (face mask)							.150
Yes	82 (67.2)	51 (61.4)	160 (74.2)	96 (71.2)	326 (66.2)	715 (68.2)	
No	40 (32.8)	32 (38.6)	56 (25.8)	39 (28.9)	167 (33.9)	333 (31.8)	

^a^ Blue-collar workers included those were service workers, sales, craft works, plat/ machine operators and assemblers, and elementary workers. ^b^ Chi-square test were performed.

For the workplace measure, only 68% respondents reported that their workplace supplied face masks to them and it was found that the availability of personal protective equipment such as face masks was lower among the groups of professional workers (38%), managerial workers (33%), and blue-collar workers (34%) in their workplace but it was not significant among the occupational groups (*P* = .150). Approximately, 14% of respondents neither had personal stocks of face masks due to accessibility issue in market nor its supply from the workplace.

###  Perceived Risk of Infection at Workplace 

Ninety-three percent were worried about being infected with COVID-19 in their workplace. Three occupational groups: blue-collar workers (97%), associate professionals (93%), and clerical support workers (91%) were significantly more worried (*P* < .001). Majority of respondents (93%, 979 of 1048) were also worried about infecting their family with COVID-19 acquired at the workplace. The level of worry about family being infected was higher than being infected themselves. Again, the 3 occupational groups of blue-collar workers (97%), clerical support workers (92%), and associate professionals (91%) were significantly more worried about family (*P* < .001) (Table 3).

**Table 3:** Perception Risk of Infection Due to COVID-19 by Occupation Groups

	**Managers and Administrator** **[n = 122]** **No. (%)**	**Professionals** **[n = 82]** **No. (%)**	**Associate Professionals** **[n = 216]** **No. (%)**	**Service/Shop Sales Workers [n = 134]** **No. (%)**	**Blue-Collar Workers** ^a^ **[n = 493]** **No. (%)**	**Total** **[n = 1048]** **No. (%)**	***P*** **Value** ^b^
Perception risk being infected from COVID-19 due to work							<.001
Not worried at all	17 (13.7)	10 (11.6)	15 (7.0)	12 (8.7)	15 (3.1)	68 (6.5)	
A bit worried	47 (38.8)	27 (33.2)	73 (33.6)	50 (37.5)	175 (35.4)	372 (35.5)	
Moderate worried	37 (30.1)	22 (26.4)	57 (26.2)	44 (32.7)	175 (35.4)	333 (31.8)	
Very worried	11 (9.3)	16 (19.3)	43 (19.9)	16 (11.5)	91 (18.5)	177 (16.9)	
Extremely worried	10 (8.2)	8 (9.6)	29 (13.3)	13 (9.6)	38 (7.7)	97 (9.3)	

Abbreviation: COVID-19, coronavirus disease 2019. ^a^Blue-collar workers included those were service workers, sales, craft works, plat/ machine operators and assemblers, and elementary workers. ^b^Chi-square test was performed.

###  Self-reported Health Outcome 

Of respondents, around two-thirds (64%) reported having a health problem in at least one of 5 dimensions of EQ-5D-5L. The highest proportion of the respondents expressed problem in anxiety/depression (55%), followed by pain/discomfort (26%), usual activities (11%), mobility (4%) and usual activities (1%); however, no significant difference (*P* = .112) was identified among the occupational groups. For the severity level of the health status in the 5 dimensions, there were statistically significant difference in 3 dimensions: usual activities (*P* = .043), pain/discomfort (*P* = .008), and anxiety/depression (*P* < .001) among the occupational groups. Blue-collar workers reported more problems in “usual activities” and “pain/discomfort,” however, associated professionals reported more problems in “anxiety/depression” (Table 4).

**Table 4:** Self-reported Health Status and HRQoL by Occupation Groups

**No. (%)**	**Managers and Administrator** **[n = 122]**	**Professionals** **[n = 82]**	**Associate Professionals** **[n = 216]**	**Service/Shop Sales Workers** **[n = 134]**	**Blue-Collar Workers** ^a^ ** [n = 493]**	**Total** **[n = 1048]**	***P *** **Value** ^b^
**Health Status**							
Any problem among EQ-5D-5L 5 dimensions							.112
Yes	82 (67.2)	57 (68.7)	152 (70.4)	80 (59.7)	304 (61.5)	674 (64.3)	
No	40 (32.8)	26 (31.3)	64 (29.6)	54 (40.3)	190 (38.5)	374 (35.7)	
EQ-5D-5L: mobility							.209
No problems	116 (95.1)	78 (94.0)	205 (94.9)	132 (98.5)	478 (97.0)	1009 (96.3)	
Slightly problems	4 (3.3)	3 (3.6)	8 (3.7)	1 (0.7)	15 (3.0)	32 (3.1)	
Moderate problems	1 (0.8)	2 (2.4)	2 (0.9)	1 (0.7)	0 (0.0)	5 (0.5)	
Severe problems	0 (0.0)	0 (0.0)	1 (0.5)	0 (0.0)	0 (0.0)	1 (0.1)	
Unable to	1 (0.8)	0 (0.0)	0 (0.0)	0 (0.0)	0 (0.0)	1 (0.1)	
EQ-5D-5L: self-care							.702
No problems	107 (88.4)	72 (86.7)	198 (91.7)	127 (94.1)	425 (86.0)	929 (88.6)	
Slightly problems	8 (6.6)	7 (8.4)	11 (5.1)	4 (3.0)	38 (7.7)	68 (6.5)	
Moderate problems	5 (4.1)	2 (2.4)	5 (2.3)	3 (2.2)	23 (4.7)	38 (3.6)	
Severe problems	1 (0.8)	1 (1.2)	2 (0.9)	1 (0.7)	0 (0.0)	5 (0.5)	
Unable to	0 (0.0)	1 (1.2)	0 (0.0)	0 (0.0)	8 (1.6)	8 (0.8)	
EQ-5D-5L: usual activities							.043
No problems	107 (88.4)	72 (87.8)	198 (91.7)	127 (94.8)	425 (86.2)	929 (88.6)	
Slightly problems	8 (6.6)	7 (8.5)	11 (5.1)	4 (3)	38 (7.7)	68 (6.5)	
Moderate problems	5 (4.1)	2 (2.4)	5 (2.3)	3 (2.2)	23 (4.7)	38 (3.6)	
Severe problems	1 (0.8)	1 (1.2)	2 (0.9)	1 (0.7)	0 (0)	5 (0.5)	
Unable to	0 (0.0)	1 (1.2)	0 (0.0)	0 (0.0)	8 (1.6)	8 (0.8)	
EQ-5D-5L: pain/discomfort							.008
No problems	97 (79.5)	61 (74.4)	155 (71.8)	114 (85.1)	349 (70.8)	776 (74.1)	
Slightly problems	21 (17.2)	19 (23.2)	59 (27.3)	19 (14.2)	106 (21.5)	225 (21.5)	
Moderate problems	3 (2.5)	2 (2.4)	2 (0.9)	1 (0.7)	30 (6.1)	38 (3.7)	
Severe problems	1 (0.8)	0 (0.0)	0 (0.0)	0 (0.0)	8 (1.6)	8 (0.8)	
Unable to	0 (0.0)	0 (0.0)	0 (0.0)	0 (0.0)	0 (0.0)	0 (0.0)	
EQ-5D-5L: anxiety/depression							<.001
No problems	47 (38.5)	32 (39.0)	75 (34.7)	59 (44.0)	258 (52.1)	471 (44.9)	
Slightly problems	55 (45.1)	38 (46.3)	96 (44.4)	54 (40.3)	175 (35.4)	418 (39.9)	
Moderate problems	15 (12.3)	9 (11.0)	29 (13.4)	16 (11.9)	38 (7.7)	107 (10.2)	
Severe problems	3 (2.5)	3 (3.7)	6 (2.8)	4 (3.0)	15 (3.0)	31 (3.0)	
Unable to	2 (1.6)	0 (0.0)	10 (4.6)	1 (0.7)	8 (1.6)	21 (2.0)	
**HRQoL**							
EQ-5D-5L Index							.012
Mean (SD)	0.895 (0.122)	0.890 (0.122)	0.884 (0.131)	0.919 (0.093)	0.898 (0.132)	0.897 (0.126)	

Abbreviations: COVID-19, coronavirus disease 2019; HRQoL, health-related quality of life; SD, standard deviation. ^a^Blue-collar workers included those were service workers, sales, craft works, plat/ machine operators and assemblers, and elementary workers. ^b^Either chi-square test, Fish exact test, Kruskal-Wallis or ANOVA were performed.

**Figure 2:**
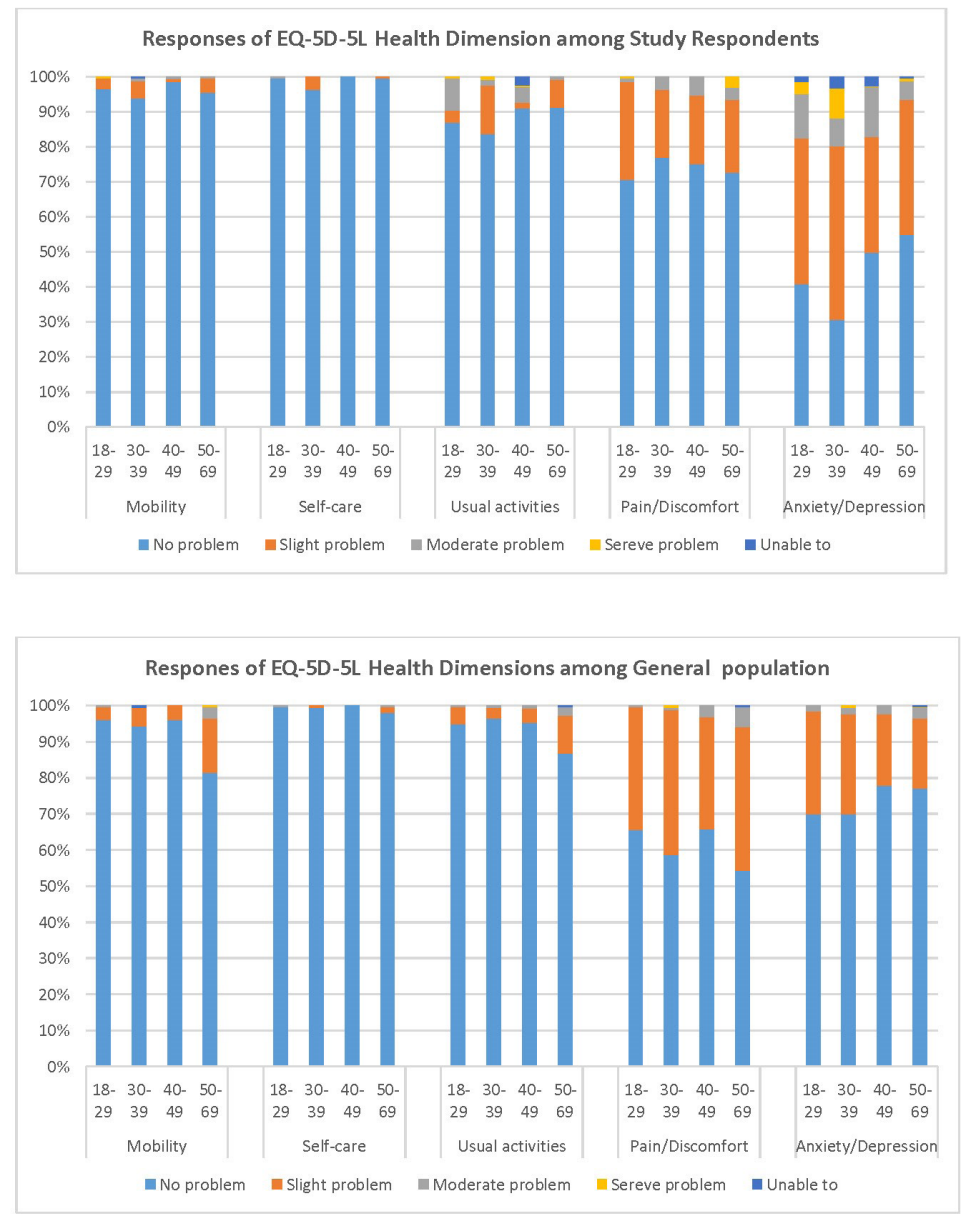
Health Status of the Respondents When Comparing With General Populations.

In responding to the HRQoL, the health index score with the application of the EQ-5D-5L HK value set was 0.897 (SD 0.126) which is significantly lower (*P* < .001) than the similar age group (aged 18-69) of the general population (0.924, SD: 0.103)^18^ and there was higher proportion reporting problems in the dimension of anxiety/depression (Figure 2). In addition, there was significant difference (*P* = .012) in HRQoL among the occupation groups in which associated professionals had the lowest health index score.

###  Mediation Analysis

 With the application of regression analysis, it shows that the perceived infection risk at workplace (worried being infected, worried infecting family) and health outcome (HRQoL-EQ5D disability, pain/discomfort, anxiety/depression) were all significantly associated with workplace policy (accessibility, transparency, comprehensiveness, timely) and measure (protective equipment supply), except the health status of mobility, self-care and social activity.

**Figure 3:**
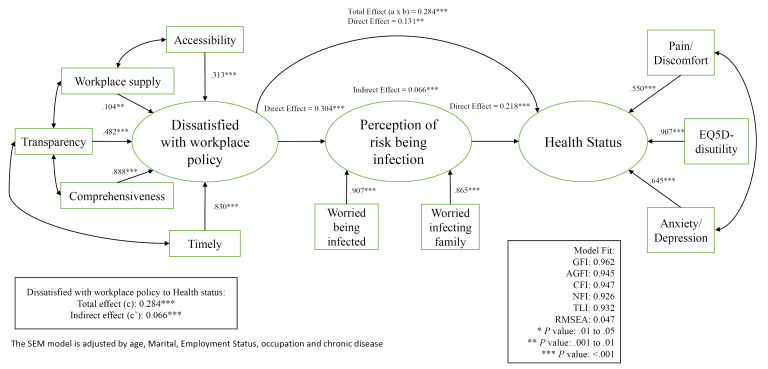
Mediation Analysis.

Path diagram (Figure 3) presented the underlying structure of the variable effects in the proposed model.Since our earlier results showed that level of health outcome was significantly associated with age, martial status, employment status, occupation and chronic disease and they were controlled for in the subsequent mediation analysis using structural equation modelling. For the statistical goodness of fit of the model, the GFI was 0.962, the AGFI was 0.945, the CFI was 0.947, the NFI was 0.926, the TLI was 0.932 and the RMSEA was 0.061 which indicated acceptable fit of the proposed model.All the path coefficients were significant and confirmed that dissatisfaction with workplace policy and measure had a total effect (c) 0.284 on lower health outcome in term of higher EQ5D-disutility, more pain/discomfort and more anxiety/depression. Workplace policy and measure had a direct effect (c’) of 0.131 on health outcome. Perception of infection risk had a direct effect of 0.218 on health outcome and partly mediated the relationship between workplace policy and measure and health outcome (indirect effect = 0.066).

###  Suggestions to Enhance Workplace Safety

 There were 168 free comments received regarding to the suggestions to enhance workplace safety at 2 levels: government policy (macro-level) and workplace policy and guideline (micro-level). For the macro-level, the top 5 suggestions were (1) full border closure (52%, 14 of 27), (2) compulsory home office for all occupations (11%, 3 of 27), (3) policy for penalizing those with hiding travel history due to the quarantine policy, quarantine, mass stock and inflation in the cost of face masks (11%, 3 of 27), (4) disseminating truth and timely information (7%, 2 of 27), and (5) providing financial subsidy for enterprises (7%, 2 of 27). Additionally, (1) promotion of stress management (51%, 72 of 141), (2) home office with technological support (16%, 23 of 141), (3) policy to reduce social distancing (14%, 20 of 141), (4) providing guideline for wearing protective measures and supply of protective measures (9%, 12 of 141), and (5) reduced workload and rescheduling timeliness of deliverables (4%, 6 of 141) were the top 5 suggestions for the micro-level

 of workplace setting.

## Discussion

 Based on our knowledge, this is the first study to explore the views on workplace safety and its impact on health status in employees during the COVID-19 epidemic. Consistent with our hypothesis, our results showed that workplace policy and measures is significantly associated with health outcome in employees. The level of dissatisfaction of infection control policy and measure at workplace are positively associated with self-reported lower health outcome, while perception of infection risk significantly mediated between the relationship between workplace policy and measure and health outcome.

 With the experience of SARS, the HK government has respond earlier during the COVID-19 pandemic; however, around one-fifth of the respondents (16%) reported that no workplace measures nor guidelines were existed in their company related to the pandemics. In addition, those who reported having workplace measures in their company were found as discrepancy in term of its comprehensiveness (36%), timeliness (38%) and transparency (63%). More than one-third of the respondents (32%) reported that the company did not supplied face masks to them in their workplace. All of these implied that the workplace measures for the pandemics was still insufficient and not in well-structured in communication.

The study found that almost all working people worried about being infected and family being infected by COVID-19 in their workplace. Concepts of risk perception mostly seemed more pragmatic than theory-based.^19^ Risk perception based on own judgement of external environment and recall memory; interestingly, our study additionally found that the level of worry about family being infected was higher than being infected themselves. Taking care of family member can be source of stress source, thus it may exaggerate the level of worry. The study also revealed that the health status and HRQoL of the respondents were worse during COVID-19 pandemic than the norms in HK.^15^ A significant high proportion reporting different levels of anxiety/depression during the COVID-19 pandemic was found in employee. This finding was similar to local study due to SARS outbreak^20^ and the study conducted in China during COVID-19 pandemic.^21^

 The findings suggested that there was a role for having workplace policy and measure in place to improve health outcome and minimize the perception of infection risk in employee. Providing comprehensive, timely and transparent information about COVID-19 pandemic and infection control guideline to protect health at workplace would benefit those who were concern about the risk of self and loved ones. The provision of protective measures in workplaces such as face masks seems to be essential factor associated with better HRQoL during the pandemics, too. The results further highlighted the availability of workplaces policy and measures as an important mean by which the tremendous stress caused by a COVID-19 pandemic can be reduced and the value of unambiguous information in reducing uncertainty.

In our survey, the respondents consistently emphasized on the importance of government policy as a key and overarching role to drive occupational safety in business including full border closure, compulsory home office, and collaborating with business sectors to formulate operational guidelines for social distancing. Singapore has been discussing the implementation and intensification of social distancing measures, including staff working at staggered hours and setting up telecommuting office to deal with the possible COVID-19 outbreak.^22^ A considerable amount of time is need for implementing social distancing measure that would restructure the organization culture and the local context of the society as the situation evolves. Without the government’s top-down policy, socially irresponsible behaviours may pose a risk to all. The COVID-19 spread likes a wave to different countries progressively; resumption of office duty and social activity may create a threat for another wave of COVID-19 globally. COVID-19 is an insidious infectious disease that may bring more serious outcomes with genetic evolution before vaccination and treatment are initiated. Therefore, workplace safety involving businesses and employers in the society is the key to long-term success in the battle of the COVID-19 pandemic.

Regarding the micro-level workplace policy at the organization, majority of respondents perceived information not being openly disclosed or lack of transparency involving any staff member with a suspected, confirmed, or close-contact cases. This information is important to alert staff to adjust their social activity accordingly and immediately implement arrangements for the workplace in terms of changes in the office layout. Respondents were worried about any non-paid leave or salary penalties for absence due to sickness or compulsory being quarantined for flu or COVID-19 which is similar to the findings of the study conducted in the United States.^23^ This study suggested that flexibility of work from home and paid sick leave granted in the workplace are important policy to protect employee’s health during a serious infectious outbreak.^23^ In the textual comments, they suggested the need for instruction on personal hygiene, wearing face masks (when, who, and how), and staff who develop flu-like symptoms to stay at home and contact health services. About one-third of respondents commented that the guideline should be updated in a timely manner as the situation evolves and highlighted the importance of providing protective resources including face masks and hand soaps when these products are unavailable in the market. Those working manually (blue-collar group) reported poor experience of having workplace policy and guidelines. It is important to strengthen the communication through mobile or other technology to disseminate the timely and update information as well as create interactive platforms for alleviating any ambiguities. In addition, the infrastructure of technology must be used to facilitate special work arrangements during epidemics. Timely updates on morbidity and mortality rates related to COVID-19 by the media; stress management techniques such as relaxation exercise, breathing, and music; and workplace layout were suggested by respondents to motivate and keep them focused on their job in an isolated and tense situation during the crisis of COVID-19 outbreak.

Despite the increase in local and global efforts to improve the infection control and prevention and awareness of personal hygiene, our findings highlight that only two-third of respondents washed hands before meals or after toileting. It is suggested that hand hygiene is more important than wearing face mask for healthy people not working in health-care setting.^24^ As indicated by previous surveys of infectious diseases during epidemics, infection control training is important to increase awareness and improve the personal hygiene performance.^22,25-28^

Our study has 2 main limitations. First, our results are based on a non-probabilistic sampling strategy. Therefore, the occupational structure was different as compared to the local working population in Hong Kong. Thus, adjusted weighting of the occupation groups was applied based on the distribution of labour force in HK.^18^ In addition, we did not recruit those aged 15–17 years due to complexity of consent seeking. Therefore, the voice of this group is not included in the survey. Our study highlighted that those working in blue-collar occupations were more worried and had worse experience of accessibility to workplace policy than did other occupational groups. The distribution of respondents in 11 industrial fields were also not proportional to the distribution in HK working population with less in ‘retail, accommodation and food services’ and ‘financing, insurance, real estate, professional and business service’ but more in ‘pubic administration, social work activities and personal service’^18^ Despite these limitations, our study provides important insight into existing shortcomings in workplace policy at macro- and micro-levels among the employee for international reference so as to mitigate the possible outbreak of COVID-19 at workplaces and address employee’s need and concern regarding to the occupational safety and health.

## Conclusion

 During the pandemic of COVID-19, workplace measures in non-healthcare settings are equally important as those in healthcare settings due to the large proportion of labour force, which may increase the risk of spread the disease in the community. This study highlighted deficiencies in the crucial aspects of guidelines for preventing the epidemic at workplace such as government’s overarching policy in terms of the macro environment involving the closing of borders, home office arrangements, and financial support for businesses; thus, timely and transparent organizational policies with operational instructions regarding protective measures and training in infection control are not evident. Effort by government should also aim to ensure the availability of protective resources and guideline in workplaces to alleviate workplace worry of infection with infectious disease as well as HRQoL. This study further hinted at the importance of health status and HRQoL during the pandemic to prevent post-traumatic stress.

## Acknowledgements

 We thank for Mr. Jack CH Lau and Ms. Amy YK Wong for their support on the data analysis and in creating the on-line survey platform and monitoring the quality of data collection.

## Ethical issues

 The study was reviewed and approved by the Survey and Behavioural Research Ethics Committee of The Chinese University of Hong Kong.

## Competing interests

 Authors declare that they have no competing interests.

## Authors’ contributions

 ELYW is the lead author and all authors designed the study and generated hypothesis. ELYW, KFH, AWLC, and PSYY analysed the data. ELYW prepared the manuscript and all authors provided substantial comments on the paper and approved the final version.
